# The Soluble Folate Receptor in Autism Spectrum Disorder: Relation to Autism Severity and Leucovorin Treatment

**DOI:** 10.3390/jpm12122033

**Published:** 2022-12-08

**Authors:** Richard E. Frye, Alison Lane, Ashley Worner, Brianna A. Werner, Patrick J. McCarty, Adrienne C. Scheck, Heidi L. Collins, Steven J. Adelman, Edward V. Quadros, Daniel A. Rossignol

**Affiliations:** 1 Rossignol Medical Center, Phoenix, AZ 85050, USA; 2 Autism Discovery and Treatment Foundation, Phoenix, AZ 85050, USA; 3 Department of Child Health, University of Arizona College of Medicine—Phoenix, Phoenix AZ 85004, USA; 4 Phoenix Children’s Hospital, Phoenix, AZ 85016, USA; 5 Creighton University School of Medicine, Phoenix, AZ 85012, USA; 6 Tulane University Medical School, New Orleans, LA 70112, USA; 7 VascularStrategies LLC, Plymouth Meeting, PA 19462, USA; 8 State University of New York—Downstate, Brooklyn, NY 11203, USA; 9 Rossignol Medical Center, Aliso Viejo, CA 92656, USA

**Keywords:** autism spectrum disorder, soluble folate receptor, folinic acid, leucovorin

## Abstract

Autism spectrum disorder (ASD) is a heterogeneous neurodevelopmental disorder with life-long consequences that affects up to 1 in 44 children. Treatment with leucovorin (folinic acid), a reduced form of folate, has been shown to improve symptoms in those with ASD and folate pathway abnormalities in controlled clinical trials. Although soluble folate binding proteins (sFBPs) have been observed in the serum of some patients with ASD, the significance of this finding has not been studied. Here, we present a cohort of ASD patients with sFBPs. These patients had severe ASD and were medically complex. Using baseline controlled open-label methodology and standardized assessments, these patients were found to improve in both core and associated ASD symptoms with leucovorin treatment. No adverse effects were related to leucovorin treatment. This is the first report of the sFBPs in ASD. This study complements ongoing controlled clinical trials and suggests that leucovorin may be effective for children with ASD who are positive for sFBPs. Further, sFBPs might be important biomarkers for treatment response to leucovorin in children with ASD. This study paves the way for further controlled studies for patients with sFBPs.

## 1. Introduction

The most recent Center for Disease Control and Prevention estimates that autism spectrum disorder (ASD) affects about 2% of children in the United States with the prevalence continuing to increase [1]. Although ASD is a behaviorally defined disorder as outlined in the Diagnostic Statistical Manual of Mental Disorders, research has linked ASD to underlying abnormalities in physiology such as immune dysfunction, oxidative stress and metabolic disorders [2]. The association of ASD with metabolic disorders is particularly compelling as abnormalities in metabolic pathways can be corrected to mitigate these deficiencies [3].

Abnormalities in folate and folate related metabolism have been consistently related to ASD in multiple studies. For example, variation in several folate genes have been linked to increased risk of ASD. Single nucleotide polymorphisms (SNPs) in methylenetetrahydrofolatereductase (MTHFR) [4], reduced folate carrier (RFC) [5] and methionine synthase (MTR) [6] alone and in combination with SNPs in other genes [7] are associated with an increased risk of developing ASD. Interestingly, a SNP in the RFC gene in the mother also increases the risk of the offspring developing ASD [8]. Abnormalities in folate-dependent one-carbon trans-methylation and trans-sulphuration pathways have repeatedly been associated with ASD [9,10] and even have the potential to be diagnostic biomarkers for ASD [11,12]. Abnormalities in gene methylation have also been linked to ASD. Indeed, methylation in specific genes [13,14] as well as altered DNA methylation in parents of children with ASD [8,15] have been reported.

Another compelling abnormality in folate metabolism which is related to ASD is the inhibition of transport of folate into the central nervous system. The relation of this abnormality to ASD was first reported in children diagnosed with cerebral folate deficiency (CFD) [16]. This abnormality is associated with dysfunction of the folate receptor α (FRα) primarily due to one of two autoantibodies that bind to the receptor protein and interfere with its function, and/or mitochondrial disease or dysfunction as the process of binding and translocating folate from the apical to basolateral side of the choroid plexus is energy dependent. Two FRα autoantibodies (FRAAs) have been described (Table 1). The blocking FRAA attaches to the folate binding site of the FRα, thereby inhibiting the ability of folate to bind to the receptor while the binding FRAA binds to other regions of the receptor and interferes with its function.

One curious phenomenon is that the standard assay cannot measure the blocking FRAA titer in certain samples. In these samples, the assay identifies more bound folate than the FRα protein that is added to bind the folate, suggesting that the sample contains folate binding proteins other than the FRα. Soluble folate binding proteins (sFBPs) have been described in the cancer literature, where membrane bound folate receptors (FRα, FRβ) and/or the non-membrane bound folate receptor (FRγ) are found in the plasma or other body fluids and are usually associated with pathological processes such as infection or malignancy [17]. However, folate binding proteins are not well characterized in ASD.

The other important aspect of disrupted folate metabolism in ASD is the fact that many individuals with ASD and FRAAs appear to respond to treatment with a specific reduced form of folate known as leucovorin (folinic acid) [16]. Although there are controlled clinical trials to support the use of leucovorin in ASD, these trials have only examined children with measurable FRAAs. Thus, the significance of sFBPs with respect to treatment is not known. The lack of knowledge regarding how to interpret sFBPs limits the ability to translate the FRAA assay into clinical practice. Thus, the aim of this study is to describe patients with sFBPs in their blood and examine the effectiveness of the use of leucovorin to treat these patients in the clinical setting.

## 2. Materials and Methods

The participants were recruited for a natural history study in ASD through the Neurodevelopmental Disorders program at Phoenix Children’s Hospital. Patients admitted to the clinic for clinical care were offered the opportunity to allow their clinical data to be used for research purposes. The protocol was approved by the Institutional Review Board at Phoenix Children’s Hospital (Phoenix, AZ, USA). Parents of participants provided written informed consent.

Patients with ASD who were seen in the clinic and consented to the study were included. Diagnosis of ASD was verified by either (i) one of the gold-standard diagnostic instruments such as the Autism Diagnostic Observation Schedule and/or Autism Diagnostic Interview-Revised (ADI-R), or (ii) documentation that the patient previously met the Diagnostic Statistical Manual of Mental Disorders Fifth Edition diagnosis of ASD along with documented functional limitations, scored within the ASD range using the Social Responsiveness Scale (SRS) and was confirmed to meet ASD criteria by the Principal Investigator (R.E.F.) who specializes in the diagnosis and treatment of children with ASD. The SRS is a standardized validated questionnaire which has good correspondence to gold-standard instruments [18,19]. In our previous clinical trial [20], method (ii) was validated by re-evaluating a portion of the participants diagnosed with method (ii) using the ADI-R and finding that their ADI-R scores fell well within the diagnostic criteria for ASD.

As part of the standard clinic intake process, patients completed a standardized medical history questionnaire, the SRS (represented as t-scores) and the Aberrant Behavior Checklist (ABC; represented as raw scores). The SRS and ABC were repeated at each visit to follow the changes in ASD related symptoms with treatment during clinical care. This standardized prospective collection of outcome data to study treatment effectiveness has been used in our previous studies [21,22]. As this data was derived from a clinical practice all decision-making and prescribing was part of standard medical care and leucovorin treatment was a standard prescription commercial form of the medication.

From the consented participants, those who underwent testing for the FRAAs were selected if their FRAA assay found that sFBPs interfered with the FRAA blocking assay. For these patients, pertinent clinical information was abstracted from the chart, including medication started and stopped at each visit and SRS and ABC scores at each visit. All dates were transformed to age in days at the visit and personal health identifiers were removed to deidentify the data for further analysis.

### 2.1. sFBP Assay

The FRAA assay was performed by Vascular Strategies (Plymouth Meeting, PA, USA) through their CLIA certified pathway. An in vitro functional blocking assay is used to measure blocking FRAAs while an enzyme-linked immunosorbent assay (ELISA) specific for binding IgG is used to measure binding FRAAs as previously described [23]. In this study, the presence of sFBPs was inferred from the blocking assay, which showed higher than expected binding of ^3^H-folic acid with no blocking activity.

### 2.2. Statistical Analysis

A mixed-model with random effects of subjects to control for repeated effects of subject level mean and variance with an autoregressive moving average covariance structure was used to analyze the data. Two separate approaches were used to determine the effect of leucovorin treatment on changes in SRS and ABC scores. The first analysis used a dichotomous variable to represent whether the individual was on leucovorin vs. whether they were not on leucovorin. The second analysis created a variable which represented the number of days of leucovorin treatment so it could be determine if symptoms change as a function of days on leucovorin.

## 3. Results

Review of the patients that underwent FRAA testing demonstrates that 14 (13%) of 110 patients had sFBPs. A description of the characteristics of these 14 patients is first provided, followed by an analysis of the change in ASD and related symptoms in 12 of the 14 patients who were treated with leucovorin.

### 3.1. Patient Characteristics

Seven of the 14 (50%) patients were positive for the binding FRAA with a mean (SD) titer of 0.68 (0.29) OD Units. Most (93%) were male and most (71%) were White while the remainder were Black, and one was of Hispanic ethnicity. Mean (SD) age was 11.0 (5.1) years of age and most (64%) demonstrated neurodevelopmental regression while the remainder reported a plateau developmental profile.

Birth history was significant for in vitro fertilization in one and prematurity (32 wk, 36 wk) in two. Of the five that had brain MRI scans, all were normal. Of the three that had EEGs, one had background slowing and one had infrequent focal right frontotemporal sharp waves. One child had Marcus Gunn Jaw Winking Phenomenon and one had a ruptured hepatoblastoma requiring repeated surgeries during infancy. Five had feeding problems, 8 had severe constipation and one had eosinophilic esophagitis. Two had severe aggressive behavior and five had significant self-injurious behavior. Five had sleep maintenance disorder, two had sleep apnea and three had insomnia.

### 3.2. Effectiveness of Leucovorin Treatment

All patients started on the standard dose of leucovorin (25 mg twice a day) but in 6 of 12 (50%), the dose of leucovorin was increased to 50 mg twice a day in order to obtain an effective response as judged by the ongoing clinical evaluation. The number of days treated with leucovorin ranged from 92 to 1022 days with an average (SD) number of 584 (333) days of treatment.

#### 3.2.1. Social Responsiveness Scale

The difference in SRS scores was compared before and after the start of leucovorin treatment. The Total score decreased by 7.4 (1.4) t-score points with leucovorin treatment [F(1,44.6) = 28.03, *p* < 0.001, Cohen’s *d*′ = 1.4]. The Awareness subscale decreased by 8.0 (1.9) t-score points with leucovorin treatment [F(1,45.9) = 18.01, *p* < 0.001, Cohen’s *d*′ = 1.1]. The Cognition subscale decreased by 8.5 (1.8) t-score points with leucovorin treatment [F(1,45.4) = 21.64, *p* < 0.001, Cohen’s *d*′ = 1.2]. The Communication subscale decreased by 8.9 (1.6) t-score points with leucovorin treatment [F(1,44.6) = 31.22, *p* < 0.001, Cohen’s *d*′ = 1.4]. The Motivation subscale decreased by 6.9 (2.1) t-score points with leucovorin treatment [F(1,44.8) = 10.91, *p* < 0.01, Cohen’s *d*′ = 0.9]. The Mannerisms subscale decreased by 4.3 (1.9) t-score points with leucovorin treatment [F(1,45.0) = 2.31, *p* = 0.03, Cohen’s *d*′ = 0.6].

**Figure 1:**
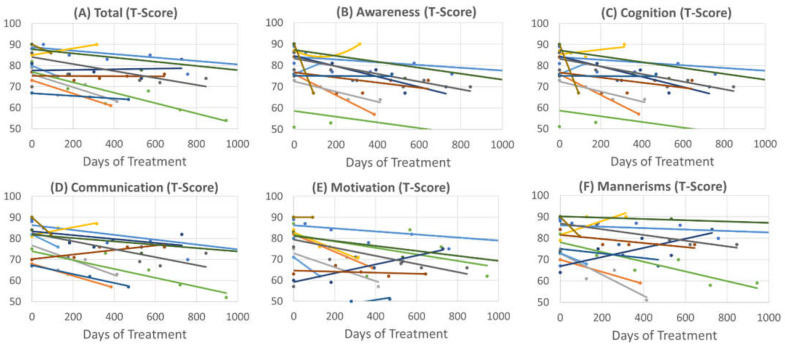
Change in the Social Responsiveness Scale (t-scores) over time with initiation of leucovorin for patients with soluble folate binding proteins: (**A**) Total Score, (**B**) Awareness, (**C**) Cognition, (**D**) Communication, (**E**) Motivation and (**F**) Mannerisms. Each color line represents a single patient.

To determine an estimate of the timing of the effect of leucovorin treatment, linear regression models were used to determine the change in SRS scores with each day of leucovorin treatment (Figure 1). The Total score decreased by 0.01 (0.002) t-score points per day on leucovorin [F(1,46.6) = 25.68, *p* < 0.001]. The Awareness subscale decreased by 0.014 (0.003) t-score points per day on leucovorin [F(1,47.2) = 19.74, *p* < 0.001]. The Cognition subscale decreased by 0.013 (0.003) t-score points per day on leucovorin [F(1,47.4) = 17.49, *p* < 0.001]. The Communication subscale decreased by 0.013 (0.003) t-score points per day on leucovorin [F(1,46.2) = 21.48, *p* < 0.001]. The Motivation subscale decreased by 0.01 (0.004) t-score points per day on leucovorin [F(1,46.1) = 8.27, *p* < 0.01]. The Mannerisms subscale decreased by 0.008 (0.003) t-score points per day on leucovorin [F(1,46.2) = 6.34, *p* = 0.02].

#### 3.2.2. Aberrant Behavior Checklist

The difference in ABC scores was compared before and after the start of leucovorin treatment. The Total score decreased by 20.2 (5.9) raw points with leucovorin treatment [F(1,44.8) = 11.92, *p* = 0.001, Cohen’s *d*′ = 0.9]. The Irritability subscale decreased by 5.7 (2.1) raw points with leucovorin treatment [F(1,44.8) = 7.56, *p* < 0.01, Cohen’s *d*′ = 0.6]. The Social Withdrawal subscale decreased by 5.8 (1.7) raw points per day with leucovorin treatment [F(1,43.1) = 11.05, *p* < 0.01, Cohen’s *d*′ = 0.9]. The Stereotypy subscale decreased by 2.2 (1.0) raw points with leucovorin treatment [F(1,46.4) = 4.56, *p* < 0.05, Cohen’s *d*′ = 0.5]. The Hyperactivity subscale decreased by 6.8 (1.6) raw points with leucovorin treatment [F(1,43.8) = 18.34, *p* < 0.001, Cohen’s *d*′ = 1.1]. Inappropriate Speech subscale did not change significantly with leucovorin treatment.

**Figure 2:**
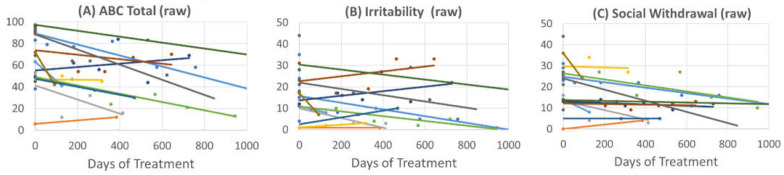

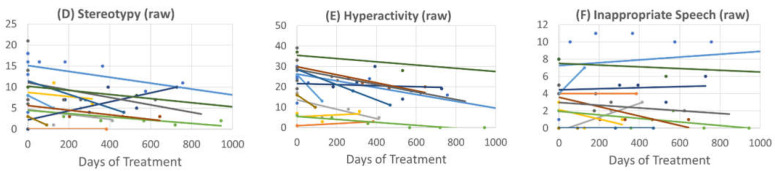
Change in the Aberrant Behavioral Scale raw score over time with initiation of leucovorin for patients with soluble folate binding proteins: (**A**) Total Score, (**B**) Irritability, (**C**) Social Withdrawal, (**D**) Stereotypy, (**E**) Hyperactivity and (**F**) Inappropriate Speech. Each color line represents a single patient.

To get an estimate of the timing of the effect of leucovorin treatment, linear regression models were used to determine the change in ABC scores with each day of leucovorin treatment (Figure 2). The Total score decreased by 0.04 (0.01) raw points per day on leucovorin [F(1,46.3) = 12.67, *p* = 0.001]. The Irritability subscale decreased by 0.008 (0.004) raw points per day on leucovorin [F(1,46.5) = 5.33, *p* < 0.05]. The Social Withdrawal subscale decreased by 0.01 (0.003) raw points per day on leucovorin [F(1,44.9) = 13.85, *p* < 0.001]. The Stereotypy subscale decreased by 0.004 (0.002) raw points per day on leucovorin [F(1,48.1) = 6.17, *p* < 0.05]. The Hyperactivity subscale decreased by 0.01 (0.003) raw points per day on leucovorin [F(1,44.6) = 20.56, *p* < 0.001]. The change in the Inappropriate Speech subscale did not change significantly with leucovorin treatment.

## 4. Discussion

This manuscript provides a description of 14 patients with ASD who were found to have a novel biomarker of folate metabolism that has not been considered in the past. This is the first study to demonstrate that 12 patients with sFBPs appear to respond to treatment with leucovorin, a treatment that has only previously been used to treat children with ASD who are FRAA positive.

Despite significant research, few validated biomarkers have been developed to assist in the diagnosis of ASD or for determining treatment response. Biomarkers of folate one-carbon metabolism are some of the most promising biomarkers for ASD, and FRAAs are one of the most promising biomarkers for determining treatment response [24]. These studies emphasize the importance of the folate pathway in ASD and point to the promise of better understanding the specific abnormalities in folate one-carbon metabolism that can predispose children to developing ASD.

The folate pathway is also critical in other neurodevelopmental disorders such as neural tube defects [25] and folate appears to be central to other diseases such as cancer [26]. However, unlike neurodevelopmental disorders, cancer treatments aim to inhibit folate metabolism [27] rather than to enhance it. sFBPs have been studied in cancer where they may have therapeutic effects [17]. While the effect of inhibiting folate metabolism in cancer may be therapeutic, it may be detrimental for the developing brain. Thus, these sFBPs may be particularly important in understanding the risk of both developing neurodevelopmental disorders and in planning treatment. Indeed, half of the participants were prescribed higher than usual doses of leucovorin in order to obtain an obvious therapeutic effect. Thus, this suggests that sFBPs may significantly disrupt folate metabolism in children with ASD, requiring more aggressive treatment.

In this clinical series, patients appeared to respond to treatment with leucovorin. The analysis suggests that treatment with leucovorin in these patients decreased the SRS score by about 7 points, a change that could potentially move the severity of the child from one category (severe, moderate, mild) to the next lower category. The decrease of the irritability subscale of the ABC was, on average, 5.7, which is an improvement consistent with the effect of anti-psychotic medications in well controlled clinical trials [28].

This study also examined the rate at which these improvements occurred. The analysis demonstrated steady, but slow, improvements with leucovorin treatment in individuals positive for sFBPs. For example, a 5.7 improvement in irritability on the ABC generally requires two years of treatment rather than the typical 12-week trials used to examine improvement with anti-psychotic medications. Similarly, two years of treatment appear to be necessary to find the 7-point improvement in the SRS. However, it should be emphasized that few other treatments are effective in improving the SRS in individuals with ASD. Additionally, leucovorin is extremely safe and well-tolerated in this vulnerable population [16] where as anti-psychotic medication can have serious long-term adverse effects [28].

This study has several limitations, most notably the small sample size and the open-labeled methodology. As this is a new area of treatment discovery in ASD, novel observations in small samples are the starting point for larger better controlled studies. The participants in this study were evaluated for FRAAs and treated with leucovorin because of the refractory nature of their disease. Indeed, the majority of patients were older, adolescents and young adults, who still had significant symptoms, many with self-injurious behavior. Thus, it is unlikely that the improvements seen were just from natural improvements. Indeed, large, well-controlled studies will be needed in the future. Lastly, sFBPs are not currently quantitatively measured. Further development of the assay to better characterize these proteins may provide a biomarker with greater fidelity for predicting treatments.

## 5. Conclusions

This manuscript reports a case-series of individuals with ASD with a previously unreported biomarker of disruption of folate metabolism. These individuals were older and refractory to previous treatments. Leucovorin treatment appeared to improve their symptoms slowly but steadily as compared to their baseline. Larger controlled studies will need to be conducted to verify these findings. Nevertheless, the observations provided in this study may be an important starting point for treatment of an important subset of individuals with ASD.

## Figures and Tables

**Table 1:** Folate Receptor Alpha Biomarkers Related to Autism Spectrum Disorder.

Biomarker	Description
Blocking Antibody	This is an antibody (any type, IgG, IgM, etc) that blocks the ability of folate to bind to the folate receptor alpha
Binding Antibody	This is an antibody (any type, IgG, IgM, etc) that binds anywhere on the folate receptor alpha
Soluble Folate Binding Proteins	These are proteins that binds folate. Their presence is inferred by an interference with the blocking assay.

## Data Availability

Data is available upon request.
